# Dynamic Changes in Host Immune Response During Crimean–Congo Hemorrhagic Fever and Severe Fever with Thrombocytopenia Syndrome in Mice

**DOI:** 10.3390/v18050504

**Published:** 2026-04-28

**Authors:** Doreswamy Kenchegowda, Brian D. Carey, Joshua Shamblin, Collin J. Fitzpatrick, Danielle L. Porier, Susan Coyne, Jeffrey Koehler, Candace D. Blancett, Christina E. Douglas, Cheryl Taylor-Howell, Aura R. Garrison, Christopher P. Stefan, Charles J. Shoemaker, Joseph W. Golden

**Affiliations:** 1 Molecular Virology Branch, Division of Virology, United States Army Medical Research Institute of Infectious Diseases, Fort Detrick, Frederick, MD 21702, USA; k.doreswamy@gmail.com (D.K.); brian.d.carey5.ctr@health.mil (B.D.C.); joshua.d.shamblin1.civ@health.mil (J.S.); collin.j.fitzpatrick.ctr@health.mil (C.J.F.); danielle.l.porier.ctr@health.mil (D.L.P.); aura.r.garrison.civ@health.mil (A.R.G.); 2 Chenega Professional and Technical Service, Chesapeake, VA 23320, USA; cheryl.l.taylor-howell.ctr@health.mil; 3 Diagnostic Systems Division, United States Army Medical Research Institute of Infectious Diseases, Fort Detrick, Frederick, MD 21702, USA; susan.r.coyne.civ@health.mil (S.C.); jeffrey.w.koehler4.civ@health.mil (J.K.); christian.e.douglas@uscg.mil (C.E.D.); christopher.p.stefan.civ@health.mil (C.P.S.); charles.j.shoemaker5.civ@health.mil (C.J.S.)

**Keywords:** Crimean–Congo hemorrhagic fever, severe fever with thrombocytopenia syndrome, interferon stimulatory genes, biomarker, chemokines, cytokines, Toll-like receptors, immune evasion

## Abstract

Crimean–Congo hemorrhagic fever virus (CCHFV) and severe fever with thrombocytopenia syndrome virus (SFTSV) are tick-borne pathogens that cause severe illness and high mortality. Early diagnosis is critical, particularly in resource-limited settings, to enable timely intervention. Host gene expression profiling offers a promising approach to identify potential biomarkers for early detection, disease staging, and logical treatment decision-making. Using a transient IFN-α/β receptor-suppressed mouse model, we performed targeted transcriptomic analysis on blood samples collected at 2, 3, and 4 days after CCHFV or SFTSV challenge. A significant increase in viral load and changes in gene expression were observed as early as two days post-challenge. CCHFV induced a progressively evolving interferon-driven response, while SFTSV triggered rapid, sustained immune activation. Affected targets included interferon-stimulated genes, chemokines, cytokines, Toll-like receptors, and genes associated with viral evasion and innate immune response. Despite shared expression patterns, unique genes were identified as potential biomarkers to distinguish between CCHFV and SFTSV infections. Differential gene expression revealed distinct immune response dynamics, with suppression of critical immune regulatory genes suggesting transcriptional signatures associated with viral evasion mechanisms contributing to disease severity. These findings provide a comparative analysis of molecular pathways and gene expression changes, offering critical insights for biomarker discovery, effective triage, and evaluation of appropriate medical intervention.

## 1. Introduction

Crimean–Congo hemorrhagic fever (CCHF) and severe fever with thrombocytopenia syndrome (SFTS) are tick-borne viral hemorrhagic fevers with remarkably similar disease characteristics and clinical presentations. Both Crimean–Congo hemorrhagic fever virus (CCHFV) and severe fever with thrombocytopenia syndrome virus (SFTSV), also known as Dabie bandavirus, belong to the *Bunyaviricetes* class but are classified into different families: Nairoviridae and Phenuiviridae, respectively. SFTSV is endemic to East Asia, whereas CCHFV is endemic to Africa, Europe, the Middle East, and Central and South Asia, including the Xinjiang Uyghur Autonomous Region of the People’s Republic of China. Both CCHFV and SFTSV are considered to be highly infectious, emerging zoonotic diseases that could cause a public health emergency. However, there are some clinical differences between the two diseases. CCHF disease manifests with a sudden onset of high fever, chills, myalgia, headache, nausea, vomiting, dizziness, and diarrhea. The later stages of disease include hemorrhagic manifestations ranging from petechia, large hematomas, subcutaneous bleeding, and hemorrhage in various organs, with a high case fatality rate of 10–40% [1,2,3,4].

SFTS patients have fever, nausea, and vomiting, often associated with elevated liver enzymes and neurological symptoms; however, severe thrombocytopenia and leukocytopenia are hallmarks of SFTS. Death in SFTSV cases is due to hemophagocytic syndrome associated with cytokine storm, disseminated intravascular coagulation, thrombocytopenia, and multiorgan failure, with a case fatality rate of 5–40% [4]. SFTS case fatality rates vary widely across East Asia, where the virus is endemic, but are typically reported to be 6–21%, with up to 75% mortality in cases complicated by hemophagocytic syndrome and multiorgan failure [5,6,7]. In addition, advanced age is an additional risk factor, as shown by a study conducted using older ferrets (>4 years), which showed severe thrombocytopenia with a high mortality rate of 93%. However, younger ferrets (<2 years) did not show SFTS-related symptoms or death [8,9]. In both cases, the disease course and outcome depend on the viral load and the balance between the host immune response mediators, and the fatal outcome is often due to a “cytokine storm” [10,11].

CCHFV infection is linked to dysregulated inflammatory responses, including upregulation of cytokines, chemokines, interferon-stimulated genes (ISGs), and TNF superfamily genes. Cytokines such as IL-6, IL-8, IL-10, IL-12, IFN-γ, MCP-1 (CCL2), and MIP-1β are critical predictors of fatal outcomes in CCHF patients [12,13,14]. Similarly, cytokine storm, along with multi-organ failure, coagulopathy, and hemophagocytosis, are major drivers of mortality in SFTSV patients [4]. Both viruses employ immunoparasitic mechanisms to manipulate host interferon (IFN) responses, underscoring the complex interplay between host immunity and viral pathogenesis [13,15,16,17,18,19,20].

To study viral pathogenesis and to develop medical countermeasures, animal models with human-like symptoms are essential. Studies show that no clinical symptoms or mortality were observed in mice with intact immune responses when infected with either CCHFV or SFTSV [12,21]. To date, a number of animal models are available to study CCHFV and SFTSV pathology, including the STAT-1 knockout mice, the interferon α/β (IFN-α/β) receptor 1 knockout (IFNAR^−/−^) mice, STAT-2 knockout mice (C57BL/6 background), and newborn mice. These models recapitulate some of the clinical features and cause severe illness and death similar to humans [21,22,23,24]. We and others have utilized an interferon suppression (IS) mouse model in which type I interferon signaling, a key component of antiviral immunity, is transiently suppressed using an antibody that blocks the IFNAR-1 subunit of IFN α/β receptor (MAR1-5A3) [25]. In CCHFV-infected mice, the IS model causes lethal disease around 5 days post challenge and recapitulates human-like signs and symptoms such as viremia, elevated liver enzymes, heightened inflammatory response, hepatocyte degeneration, and necrosis in C57BL/6 mice [12,26,27,28]. The IS model also results in lethal disease in C57BL/6 mice during SFTSV infection, with clinical manifestations including loss of weight, altered hematological parameters (thrombocytopenia), cytokine storm, and pathological changes in liver, small intestine, and spleen [21].

Understanding the molecular targets and pathways underlying CCHFV and SFTSV infection is a prerequisite to developing effective antivirals, vaccines, and diagnostic techniques. Here, we examined host immune responses to CCHFV and SFTSV using targeted transcriptomics on the NanoString platform. We aim to characterize the host transcriptional dynamics from disease onset to severe progression using a murine IS model, identifying potential biomarkers to assess disease severity and distinguishing CCHF from SFTS. By analyzing immune gene expression profiles, we explore pathways driving viral pathogenesis and immune evasion, focusing on differential gene expressions linked to disease progression. These insights are crucial for assessing disease severity and guiding the development of medical countermeasures to improve clinical outcomes.

## 2. Materials and Methods

### 2.1. Virus Strain and Reagents

All experiments involving replicating viruses were conducted under biosafety level 4 (BSL-4) containment at the United States Army Medical Research Institute of Infectious Diseases (USAMRIID), Fort Detrick, MD, USA, following approved standard operating procedures. The CCHFV strain Afg09-2990 [29] and the SFTSV strain USAMRIID-HLP23_VE6 [30] were sourced from the USAMRIID viral repository. Anti-Mouse IFNAR-1 (Catalog number 1188, MAR1-5A3) was procured from Leinco Technologies, Inc. (St. Louis, MO, USA).

### 2.2. Mouse Studies

All animal studies were conducted in Animal Biosafety Level 4 (ABSL-4) laboratories at USAMRIID. C57BL/6N mice (8–10 weeks old) were obtained from Charles River Laboratories (Frederick, MD, USA) and acclimatized in the ABSL-4 facility for at least five days prior to virus challenge. Mice were randomly assigned to two groups of 15. On day −1, animals were injected intraperitoneally (i.p.) with MAR1-5A3 (2.0 mg/mouse) to transiently suppress interferon responses. On day 0, animals were infected i.p. with either CCHFV (1 × 10^2^ pfu/mouse, n = 15) or SFTSV (1 × 10^5^ pfu/mouse, n = 15). On day +1, a second dose of MAR1-5A3 (0.5 mg/mouse) was administered i.p. Control groups (n = 5) received MAR1-5A3 but were uninfected. Animals were monitored at least once daily for signs and symptoms, humanely euthanized (n = 5 per time point) on days +2, +3, and +4 post-infection. Blood samples were collected by cardiac puncture.

### 2.3. Virus Copy Number Analysis

Whole blood samples were inactivated using a 3:1 ratio of TRIzol™ LS Reagent to whole blood (ThermoFisher, Waltham, MA, USA). Total nucleic acids were extracted using the EZ1/2 Virus Mini Kit v2.0 (Qiagen, Venlo, The Netherlands) on the EZ1 Advanced XL robot (Qiagen), following the manufacturer’s instructions. RT-qPCR was performed using equal amounts of RNA by TaqPath™ 1-Step RT-qPCR Master Mix, CG (ThermoFisher) on the QuantStudio DX (ThermoFisher). Primers and probes targeted the nucleocapsid protein gene of CCHFV (Forward: 5′-GGAATGGTGTAGGGAATTTG-3′, Reverse: 5′-CAAGGTGGGTTGAAAGC-3′, Probe: 6FAM 5′-CAAAGGCAAGTACATAAT-3′ MGNBQ) and SFTSV (Forward: 5′-GCTGGGAAGGRGTTTATAATGATGC-3′, Reverse: 5′-CGGTCTGRTTGCTGTCAAGGA-3′, Probe: 6FAM 5′-AGAATCAATTGGATAAGYG-3′ MGNBQ). Reference material was quantified using the QIAcuity Digital PCR System and QIAcuity One-Step Viral RT-PCR Kit (Qiagen) with the same RT-qPCR assay. Equal amounts of RNA Samples were run in technical triplicates, and viral concentrations were calculated using a standard curve generated from quantified reference material included in each run. Viral copy numbers were expressed as log_10_ Genomic Copy Equivalents/reaction (GCE/rxn). Samples outside the standard curve range were extrapolated to estimate copy numbers.

### 2.4. RNA Extraction from Blood

Whole blood samples were inactivated using a 3:1 ratio of TRIzol LS (ThermoFisher Scientific, Waltham, MA, USA) and stored at −80 °C until RNA extraction. RNA for NanoString analysis was manually extracted using the miRNeasy kit (QIAGEN, Venlo, The Netherlands) according to the manufacturer’s instructions, with minor modifications. The TRIzol LS/whole blood mixture was placed in a Phasemaker phase separation tube (ThermoFisher Scientific) and centrifuged at 12,000× *g* for 15 min at room temperature. The top aqueous phase was mixed with absolute ethanol, transferred to a miRNeasy kit column, and processed per the manufacturer’s protocol. Total RNA was quantified using a Qubit Fluorimeter (ThermoFisher Scientific).

### 2.5. Targeted Transcriptomic Analysis

For host transcriptomic analysis, we used the nCounter^®^ mouse host response panel (Bruker Inc., Bothell, WA, USA), comprising 785 genes targeting host responses to infectious diseases. Hybridization buffer (70 µL) was combined with 42 µL of reporter code set to prepare a master mixture. An aliquot of the master mix (8 µL) was added to separate tubes containing 50 ng of extracted RNA from mouse blood and 2 µL of the capture code set, followed by thorough mixing. The hybridization mixture was incubated at 65 °C for 17 h and then held at 4 °C until analysis. Samples were analyzed using the NanoString SPRINT™ Profiler analysis system (Bruker), and data were extracted as RCC files for downstream analysis.

### 2.6. Differential Gene Expression Analysis

Normalized count data were generated using NanoString criteria and extracted from RCC files via the ROSALIND Bioinformatics software suite (ROSALIND Bio, San Diego, CA, USA) [31,32,33,34,35,36]. ROSALIND^®^ applies the nCounter^®^ Advanced Analysis protocol, normalizing counts within a lane by dividing them by the geometric mean of normalizer probes from the same lane. Housekeeping probes used for normalization were selected using the geNorm algorithm, implemented in the NormqPCR R library [36]. Differentially expressed genes were identified based on a filter linear threshold of ±2.0-fold change and an adjusted *p*-value < 0.05, relative to whole blood samples from unchallenged mouse groups.

### 2.7. Quantitative Real-Time PCR (RT-qPCR)

RNA extracted from blood using the RNeasy kit (Qiagen) was quantified using a NanoDrop spectrophotometer, and equal amounts of RNA were used for RT-qPCR analysis. One-step RT-qPCR was performed using the RNA-to-CT™ 1-Step Kit and inventoried TaqMan assays (ThermoFisher). Data were analyzed using the comparative Ct method, normalized to GAPDH, and reported as fold-change relative to the uninfected control group.

### 2.8. Pathway Analysis

Genes exhibiting a fold change greater than 2 and a *p*-value less than 0.05 were analyzed using iDEP 2.0 (https://bioinformatics.sdstate.edu/idep/ accessed on 4 April 2025) [37], utilizing data normalized to internal controls from NanoString analysis. Pathway enrichment analysis was conducted using ShinyGO 0.85.1, a graphical gene-set enrichment analysis tool (https://bioinformatics.sdstate.edu/go/ accessed on 6 June 2025). Enriched pathways were identified based on Gene Ontology (GO) Biological Process terms and visualized as heat maps generated using GraphPad Prism (Version 10). Additional pathway analysis was performed using “REACTOME”, a manually curated and peer-reviewed pathway database, and enriched pathways were ranked based on their significance.

## 3. Results

### 3.1. Viremia in Blood

The experimental design aimed to capture the dynamic changes in gene expression associated with the host immune response during CCHFV and SFTSV infections. To achieve this, we used a mouse model in which type I interferon (IFN) signaling was blocked by depleting the IFN-α/β (IFNAR-1 subunit) using MAR1-5A3 antibody. Adult C57BL/6 mice were challenged with either PBS (control), CCHFV strain Afg09-2990 (100 pfu), or SFTSV strain USAMRIID-HLP23_VE6 (100,000 pfu). In order to compare gene expression in lethal models of CCHF and SFTS, different doses of virus were used to achieve a uniformly lethal disease with the lowest possible dose [12,27,28,29]. Total RNA was extracted from blood samples collected at three critical time points: day 2 (early stage), day 3 (mid stage), and day 4 (late stage) post-challenge. Post-challenge time points were selected to align with the disease progression in the mouse model, capturing key stages of viral replication and host immune response.

**Figure 1:**
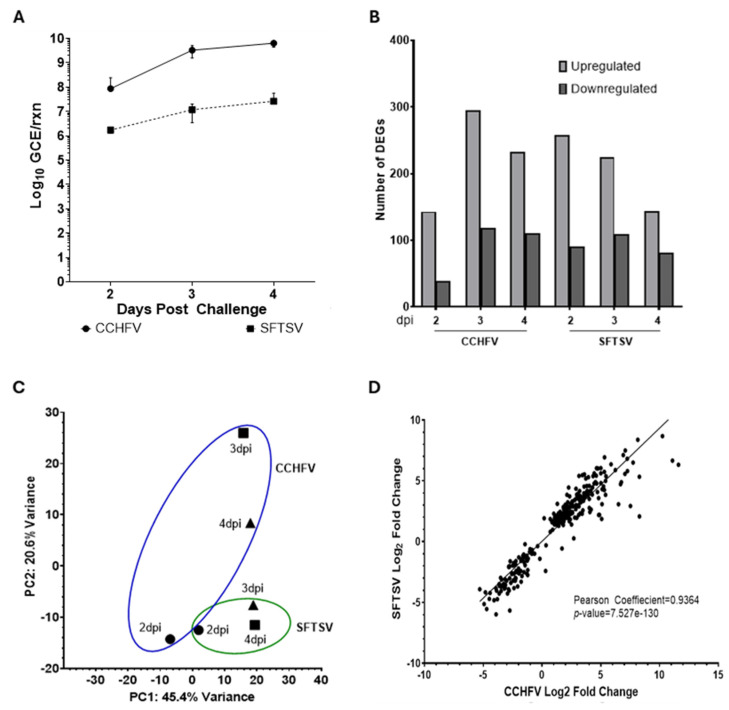
Viral titer determination and differentially expressed genes during CCHFV and SFTSV infection. Mice pretreated with MAR1-5A3 antibody were challenged with CCHFV (1 × 10^2^ pfu/mouse) and SFTSV (1 × 10^5^ pfu/mouse). Blood samples were collected on 2, 3, and 4 days post-infection (dpi). (**A**) Viral titer in blood samples determined using RT-qPCR and expressed as log_10_ genomic copy equivalents/reaction (GCE/rxn). (**B**) Number of upregulated and downregulated genes at 2, 3, and 4 dpi identified by targeted NanoString analysis of RNA. (**C**) Principal Component Analysis plot showing variance in data across two components, PC1 and PC2. (**D**) Scatter plot showing the correlation between log_2_ fold changes in gene expression across CCHFV and SFTSV at 2, 3, and 4 dpi.

Viral copy numbers in blood samples from mice infected with CCHFV and SFTSV were quantified at days 2, 3, and 4 post-challenge using RT-qPCR (Figure 1A). In CCHFV-infected mice, viral RNA increased progressively, starting at day 2 (8.7 × 10^7^ genomic copy equivalents/reaction, GCE/rxn), rising by nearly 1 log by day 3, and peaking at day 4 (6.4 × 10^9^ GCE/rxn), indicating an active and sustained viral replication over time. SFTSV-infected mice exhibited high viral titer as early as day 2 (1.7 × 10^6^ GCE/rxn), increasing by approximately one log on day 3 and remaining consistent through day 4 (1.2 × 10^7^ and 2.6 × 10^7^ GCE/rxn, respectively), suggesting early replication followed by stable viral presence (Figure 1A). These findings highlight distinct differences in titer between CCHFV and SFTSV, with a lower viral burden in SFTSV-challenged animals, even with a significantly higher dose. Regardless of titer differences, both viruses show similar viral kinetics throughout the study.

### 3.2. Comparison of Differentially Expressed Genes (DEGs) Using Transcriptomics

Targeted transcriptomic analysis was conducted on total RNA extracted from mice to identify key gene expression changes associated with the host immune response to CCHFV and SFTSV infections. Using the nCounter^®^ Host Response Panel, we assessed the expression levels of approximately 750 genes involved in host immune response pathways. The number of DEGs in blood samples collected from mice at 2, 3, and 4 days post-infection (dpi) with CCHFV and SFTSV was identified through NanoString analysis, compared to RNA transcript levels in PBS-treated control mice. DEGs were defined as those exhibiting a log_2_ fold change greater than two (upregulated) or less than negative two (downregulated), with a statistical significance threshold of *p* < 0.05 (Figure 1B).

For CCHFV-infected mice, the number of DEGs increased over time, with the highest number observed on day 3 (295-upregulated; 118 downregulated genes). In contrast, SFTSV-infected mice showed a different pattern, with a relatively higher number of gene expression changes present on day 2 (258-upregulated; 91 downregulated genes) followed by a slight decrease on days 3 and 4, suggesting an early and sustained immune response. Overall, the distinct temporal changes in gene expression response during CCHFV and SFTSV infection indicate differential host responses and disease progression between the two viruses (Figure 1B). The Principal Component Analysis (PCA) plot reveals distinct separation between the gene expression profiles of CCHFV-infected mice (blue cluster) and SFTSV-infected mice (green cluster), indicating significant differences in host immune responses to the two viruses. The CCHFV samples show a broader distribution across the principal components, suggesting more dynamic and progressive changes in gene expression over time. In contrast, the SFTSV samples form a tighter cluster, reflecting a more consistent and stable gene expression pattern across the three time points (Figure 1C).

The comparison of log_2_ fold changes at 4 dpi reveals a strong positive correlation in host response genes during CCHFV and SFTSV infections, indicating shared immune response pathways between the two viruses (Pearson coefficient = 0.9363). Genes with positive log_2_ fold change values are upregulated in SFTSV compared to CCHFV, whereas genes with negative values are downregulated in SFTSV relative to CCHFV (Figure 1D). This correlation suggests overlapping mechanisms in the host immune response to both infections. Further, pathway analysis could provide insights into similarities and differences in the biological processes impacted by each virus.

**Figure 2:**
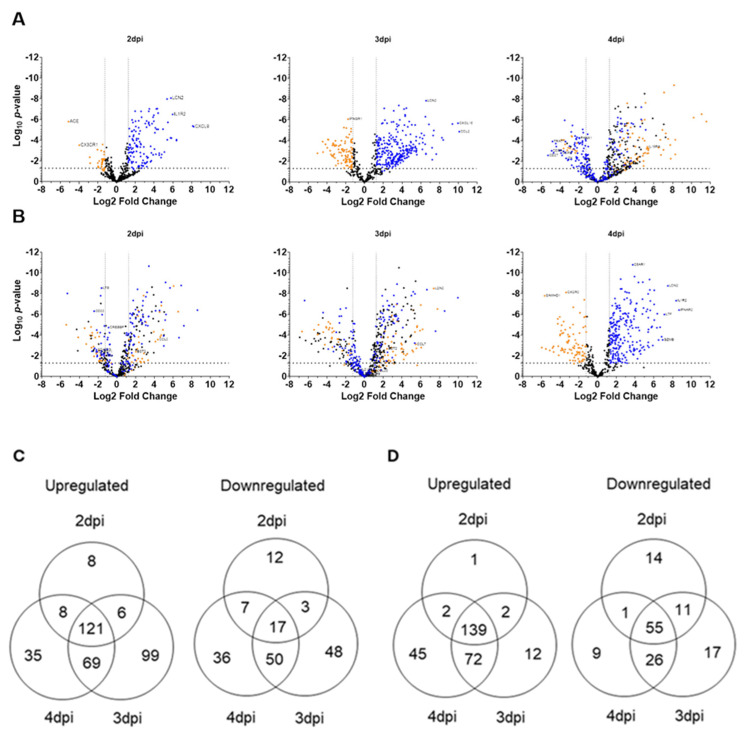
Volcano plots show differentially expressed genes during CCHFV and SFTSV infection. RNA extracted at 2, 3, and 4 dpi was subjected to NanoString analysis, showing log_2_ fold changes in downregulated (orange dots) and upregulated (blue dots) genes during CCHFV (**A**) and SFTSV (**B**). Dotted lines indicate significance cutoffs. Venn diagrams showing the number of unique and overlapping differentially expressed genes during CCHFV (**C**) and SFTSV (**D**) infection, where each ellipse represents 2, 3, and 4 dpi.

CCHFV infection resulted in a moderate number of genes upregulated (blue dots) and downregulated (orange dots), indicating an early immune response at 2 dpi (Figure 2A). A substantial increase in the DEGs was observed at 3 and 4 dpi, reflecting host response consistent with higher viral load during these timepoints. In SFTSV, a large number of genes were significantly upregulated as early as 2 dpi, indicating an early and rapid host response (Figure 2B). By 3 dpi, the number of DEGs remains relatively stable, with consistent patterns of gene regulation. Minimal changes in the number of DEGs at 4 dpi compared to earlier time points, suggesting a sustained but less dynamic immune response. Overall, CCHFV infection shows a progressive increase in DEGs over time, while SFTSV exhibits an early and sustained response, indicating a unique and dynamic host response for each virus.

Temporal changes and the distribution of DEGs with more than 2-fold changes during CCHFV and SFTSV infections are illustrated as Venn diagrams (Figure 2C and Figure 2D, respectively). In CCHFV infection, a substantial number of genes are unique to each time point, particularly at 3 and 4 dpi. Additionally, a large overlap of genes between 3 and 4 dpi suggests shared host immune responses during the later stages of CCHFV infection (Figure 2C). In contrast, SFTSV infection exhibits a higher degree of overlap in both upregulated and downregulated genes across all three time points, indicating a rapid and sustained activation of the host immune response, with fewer unique genes compared to CCHFV (Figure 2D). A comparison of common upregulated and downregulated genes between the two viruses at 2, 3, and 4 dpi reveals substantial overlap, which increases over time, suggesting convergence of host immune responses at later stages of infection (Supplementary Figure S1). However, each virus retains a subset of unique genes, reflecting host responses specific to each virus.

### 3.3. Validation of NanoString Data

**Figure 3:**
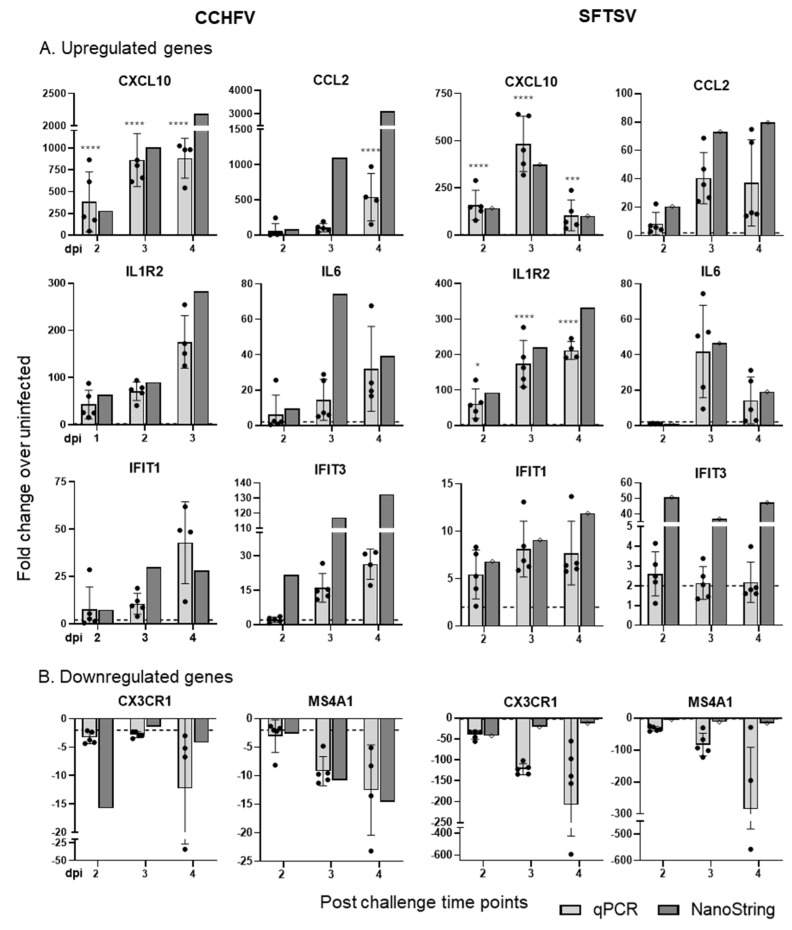
Validation of NanoString data using RT-qPCR. Upregulated and downregulated genes from blood samples collected at 2, 3, and 4 dpi were analyzed using RT-qPCR with the ddCT method. GAPDH was used as an endogenous control. The RT-qPCR results are presented as individual data points relative to uninfected mice. Values are shown as mean ± SD (n = 4–5 per group). * = *p* < 0.05, *** = *p* < 0.0005, **** = *p* < 0.0001. For comparison, NanoString results for each gene are presented as average fold change compared to uninfected mice (n = 4–5 mice per group). dpi: days post-infection.

To verify the NanoString results, we analyzed a subset of the DEGs using the TaqMan RNA-to-CT One-Step Kit and inventoried gene expression assays through quantitative Real-Time PCR (RT-qPCR) (Figure 3). In CCHFV-infected mice, the chemokines and cytokine receptors, *Cxcl10*, *Ccl2*, and *Il1r1,* exhibited a time-dependent increase in expression, with consistent results observed across both NanoString and RT-qPCR methods. Similarly, in SFTSV-infected mice, *Ccl2* and *Il1r2* levels increased over time, while *Cxcl10* in SFTSV and *Il6* in both groups peaked at 3 dpi and declined by 4 dpi. The expression patterns of interferon-stimulatory genes (ISGs), *Ifit1* and *Ifit3*, also aligned with NanoString results. Among the downregulated genes, *Cx3cr1* and *Ms4a1* were consistently downregulated in both CCHFV- and SFTSV-infected mice, as confirmed by both NanoString and RT-qPCR analyses. There are some differences in expression levels between RT-qPCR and Nanostring analysis of the same genes and time points; however, the results trend in a consistent fashion, showing that changes in gene expression can be shown with either method. Additionally, Nanostring is an extremely sensitive tool that can detect copy numbers in the single digits, which can explain some of the differences between Nanostring and RT-qPCR results. In general, these findings demonstrate that RT-qPCR results mostly corroborate the NanoString data, validating the differential gene expression patterns and supporting further investigation of genes identified through NanoString analysis.

### 3.4. Pathway Enrichment Analysis

**Figure 4:**
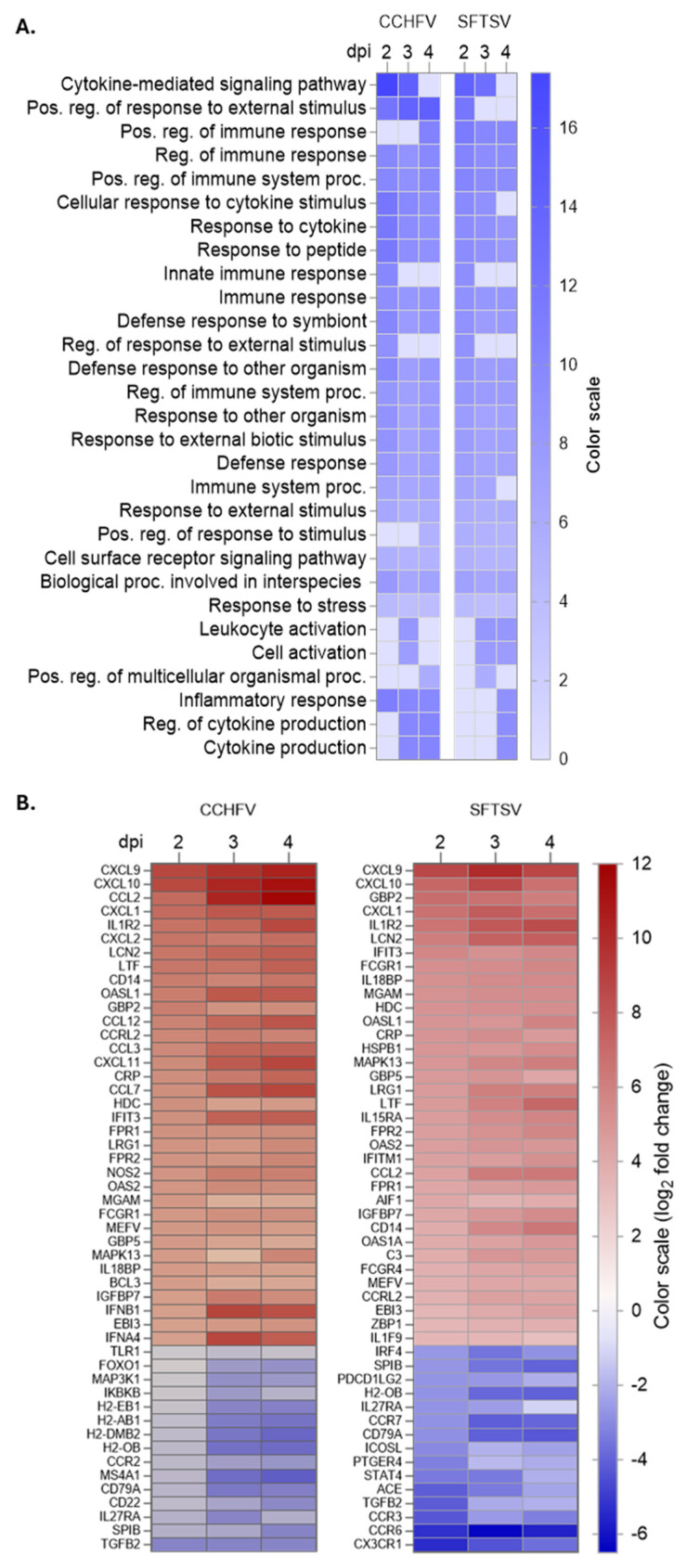
Pathway enrichment analysis. (**A**) Most enriched Gene Ontology (GO) terms based on biological processes identified by ShinyGO using differentially expressed genes at 2, 3, and 4 dpi with CCHFV or SFTSV. Genes with more than 2-fold changes (log_2_) and *p* < 0.05 were used for analysis. The color scale indicates fold enrichment of individual pathways. (**B**) Heat map of the top 50 upregulated (red squares) and downregulated (blue squares) genes during CCHFV or SFTSV infection at 2, 3, and 4 dpi (*p* < 0.05). dpi: days post-infection.

The pathway enrichment analysis was performed using Gene Ontology (GO) terms based on biological processes (ShinyGO 0.85.1). Genes with more than a 2-fold change at 2-, 3-, and 4 days post-infection (dpi) were included for analysis. Early CCHFV infection (2 dpi) was characterized by significant enrichment in pathways such as “cytokine-mediated signaling pathway” (17.4-fold), “positive regulation of response to external stimulus” (12.1-fold), and “cellular response to cytokine stimulus” (11.9-fold), indicating an early activation of cytokine signaling and immune responses. By mid-infection (3 dpi), pathways such as “positive regulation of immune response” (10.5-fold) and “regulation of cytokine production” (9.9-fold) became prominent, reflecting heightened immune activation. Later timepoint (4 dpi) showed continued enrichment in pathways like “positive regulation of immune system processes” (9.6-fold) and “cytokine production” (10.2-fold), suggesting sustained immune responses (Figure 4A).

In SFTSV-infected mice, early infection (2 dpi) also showed enrichment in pathways such as “cytokine-mediated signaling pathway” (14.2-fold) and “positive regulation of response to external stimulus” (11.9-fold), similar to CCHFV. However, SFTSV demonstrated a more sustained activation of immune-related pathways across all time points, with consistent enrichment in “positive regulation of immune response” (9.9-fold) and “regulation of immune system processes” (9.3-fold) at 3 and 4 dpi. Notably, pathways such as “inflammatory response” (8.9-fold), “leukocyte activation” (8.2-fold), and “cytokine production” were uniquely enriched in SFTSV at later stages, indicating a distinct immune activation profile compared to CCHFV (Figure 4A). We conducted additional pathway analysis using “REACTOME” to examine genes upregulated and downregulated by more than two-fold. The upregulated pathways predominantly included immune response, cytokine and chemokine signaling, and Toll-like receptor (TLR) pathways, reflecting robust activation of antiviral defenses. Conversely, downregulated pathways involved the TLR cascade, chemokines and chemokine receptors, as well as adaptive and innate immune response genes, suggesting virus-mediated host immune-evasion strategies (Supplementary Tables S3 and S4). These results highlight the dynamic and time-dependent progression of host immune response pathways during CCHFV and SFTSV infection.

In CCHFV-infected mice, the DEGs include key cytokines, chemokines, and interferon-stimulated genes (ISGs) such as *Cxcl9*, *Cxcl10*, *Ccl2*, *Oasl1*, *Gbp2,* and *Ifit3*, which are among the top 50 upregulated or downregulated genes and are involved in antiviral responses and immune activation (Figure 4B). Downregulated genes include *Tgfbb2* and *Ms4a1*, which are associated with immune cell signaling and regulation. These patterns reflect the dynamic immune response to CCHFV, characterized by progressive activation of antiviral pathways and suppression of certain immune regulatory genes. In SFTSV-infected mice, the upregulated genes also include cytokines and ISGs such as *Il6*, *Ccl2*, and *Cxcl10*, indicating rapid and sustained activation of immune responses. However, the expression of certain genes peaks earlier (e.g., *Il6* at 3 dpi) and declines by 4 dpi, suggesting a more transient immune activation compared to CCHFV. Downregulated genes in SFTSV include those involved in immune regulation, similar to CCHFV. These results highlight the distinct molecular signatures of host responses to CCHFV and SFTSV, with CCHFV showing a progressively evolving response and SFTSV eliciting a rapid and sustained activation of immune pathways (Figure 4B).

### 3.5. Unique Molecular Signatures in CCHFV and SFTSV

During CCHFV infection, interferon-related genes such as *Ifnb1*, *Ifna4*, *Ifna2*, and *Ifnl2/3* are highly upregulated, particularly at 3 dpi and 4 dpi, with fold changes exceeding 300-fold for *Ifnb1* at 3 dpi, suggestive of a robust activation of type I and III interferon pathways, which are critical for antiviral defense. However, these genes were not induced in SFTSV infection. Other upregulated genes during CCHFV infection include *Defa1*(defensin alpha), which plays a role in innate immunity, and *Cxcl3*, a chemokine involved in neutrophil recruitment. Downregulated genes such as *Sting* (a key regulator of interferon production) and *Ccr2* (a chemokine receptor involved in monocyte migration) suggest potential viral strategies to suppress specific immune pathways and evade host defenses (Table 1). During SFTSV infection, genes such as *Ccr5*, *Cxcl12*, and *Vwf* are consistently upregulated across all time points, which can be indicative of sustained activation of immune cell recruitment, endothelial damage, and tissue repair mechanisms, respectively. Upregulated genes like *Samhd1* are known to act as a restriction factor for viral replication, and *Casp1* typically triggers inflammation and apoptosis. Downregulated genes such as *Ptger4* (prostaglandin receptor) and *Atm* (DNA damage response regulator) suggest suppression of inflammatory and repair pathways, potentially contributing to immune dysregulation. Notably, *Cd40lg* ligand binds to its receptor, required to trigger survival signals, and *Bcl2*, an anti-apoptotic protein induced by *Cd40lg*, is significantly downregulated, indicating alterations in survival pathways (Table 1). These unique genes have potential as biomarkers for disease monitoring and to distinguish CCHFV from SFTSV infection.

### 3.6. Comparison of Interferon Stimulatory Genes (ISGs)

Differential expression dynamics of ISGs in mice infected with CCHFV and SFTSV are known to play a significant role in antiviral immunity, immune regulation, and host-pathogen interactions (Table 2). Many ISGs, such as *Oasl1*, *Oas*, *Oasl1a,* and *Oas*, essential for degrading viral RNA and limiting viral replication, are highly upregulated in CCHFV with fold changes peaking at 3 and 4 dpi. *Ifit3*, *Ifit1*, and *Ifi44* exhibit substantial upregulation, with *Ifit3* reaching 117-fold at 3 dpi and *Ifi44* peaking at 213.8-fold at 4 dpi. These genes are critical for inhibiting viral replication and degrading viral RNA. Downregulated genes, such as *Stat4* and *Irf4*, suggest potential transcriptional signatures associated with viral evasion mechanisms targeting immune signaling pathways to suppress adaptive immune response.

In SFTSV infection, type I interferon genes also show significant upregulation, but the response is more rapid and sustained across all time points. *Oasl1* and *Oas2* are consistently upregulated, with *Oasl1* peaking at 55.8-fold at 4 dpi. ISGs, such as *Ifitm1*, *Ifitm3*, and *Gbp2,* exhibit strong upregulation, with *Ifitm1* reaching 36.5-fold at 4 dpi, indicating robust activation of antiviral restriction factors that block viral entry and replication. *Stat4*, *Irf4,* and *Trim6* and *Trim26*, which are required for JAK-STAT signaling and modulating interferon responses, were downregulated at different time points, suggesting active suppression of interferon signaling. In addition, endoplasmic reticulum-associated antiviral effector, *Rsad2*, also known as viperin, was marginally downregulated in both CCHFV and SFTSV infection. A robust and time-dependent increase in *Mx-1* was observed in CCHFV-infected mice; however, the increase in SFTSV-infected mice was minimal compared to CCHFV. *Mx-1*, which encodes a GTPase that inhibits viral replication, was strongly induced in CCHFV compared to SFTSV, suggesting a strong response to inhibit CCHFV replication, and an increase in its expression indicates elevated antiviral defense. These observations suggest that CCHFV and SFTSV actively suppress genes involved in interferon signaling and the host’s ability to detect infection and direct antiviral responses.

### 3.7. Differential Expression of Chemokines and Cytokines

Comparison of gene expression changes of chemokines and cytokines in CCHFV and SFTSV-infected mice reveals significant differences in genes relevant to innate and adaptive immune responses (Table 3). Notably, *Cxcl9* and *Cxcl10* exhibit markedly higher expression in CCHFV, with *Cxcl9* peaking at 1208-fold and *Cxcl10* at 2184-fold at 4 dpi, compared to lower peaks in SFTSV (1000-fold and 373-fold, respectively). This suggests a more robust inflammatory and chemokine response in CCHFV, potentially contributing to severe vascular leakage and immune dysregulation, making these genes promising potential biomarkers to assess disease severity and to assess the efficacy of medical countermeasures. *Ccl2*, also known as monocyte chemoattractant protein (*Mcp-1*), expression was dramatically elevated in CCHFV, peaking at 3108-fold at 4 dpi, while SFTSV shows much lower levels, indicating a stronger monocyte recruitment and cytokine storm. *Cxcl1*, *Cxcl2,* and *Cxcr2* levels were increased as early as 2 dpi, indicating exaggerated inflammatory response. *Cxcl1* and *Cxcl2* bind to *Cxcr2* receptor expressed on vascular endothelial cells, increasing vascular permeability and hemorrhagic manifestations. Overproduction of these chemokines in SFTSV-infected mice could contribute to increased inflammation, which is a hallmark of SFTSV infection. Interleukin-related gene, *Il1r2,* known to modulate inflammatory response, was upregulated significantly in a time-dependent manner in both CCHFV and SFTSV infections. Furthermore, key inflammatory cytokines, *Il6* and *Tnf*-α are elevated in CCHFV and SFTSV, correlating with severe disease symptoms; however, SFTSV shows relatively subdued expression. Overall, the excessive inflammatory response in CCHFV aligns with its clinical presentation as a hemorrhagic fever, while the relatively lower response in SFTSV reflects its distinct pathogenesis from CCHFV. In summary, the differential expression of chemokines and cytokines highlights distinct immune response dynamics between CCHFV and SFTSV infections. CCHFV induces a progressively evolving immune response, with peak activation at later stages (3–4 dpi), reflecting delayed but robust immune activation. In contrast, SFTSV elicits a rapid and sustained immune response, with earlier peaks in chemokine and cytokine expression (Table 3); however, the magnitude of many of its DEGs generally appeared lower than that elicited by CCHFV.

## 4. Discussion

Using a well-established murine interferon suppression (IS) model [12,27,28], we conducted comprehensive targeted transcriptomic analysis to investigate the complex relationship among serum viral load, proinflammatory cytokines, chemokines, interferon-stimulated genes (ISGs), and TLRs during CCHFV and SFTSV infections. Viral copy numbers quantified using RT-qPCR revealed similar replication kinetics between CCHFV and SFTSV infections; however, even with a 1000-fold higher dose, SFTSV replicated to lower levels. The NanoString analysis using a murine panel with approximately 750 immune-related genes revealed distinct temporal and virus-specific patterns of DEGs during CCHFV and SFTSV infections. CCHFV infection demonstrated a progressive increase in DEGs over time, with the highest number observed at 3 dpi, indicating a dynamic and evolving immune response. In contrast, SFTSV infection elicited an early and sustained response, with a peak in DEGs at 2 dpi followed by relatively stable expression patterns at later time points. Pathway enrichment analysis using Gene Ontology (GO) terms further highlighted these differences, with CCHFV showing progressive activation of cytokine signaling and immune pathways, while SFTSV exhibited consistent enrichment in immune-related pathways and unique activation of inflammatory and leukocyte-related processes at later stages. PCA plot revealed significant differences in gene expression profiles, with CCHFV showing broader temporal variability and SFTSV exhibiting a more consistent response. The reliability of the NanoString results was confirmed by analyzing randomly selected genes using RT-qPCR assays.

The innate immune response, mediated by pattern recognition receptors such as TLRs and RIG-I-like receptors, activates signaling pathways that produce cytokines, chemokines, and interferons (IFNs), which subsequently induce interferon-stimulated genes (ISGs) [38]. Previously, we and others showed a detailed analysis of interferon-stimulatory gene (ISG) expression dynamics in mice and NHPs infected with CCHFV, highlighting significant changes over time and offering insights into the host antiviral response [12,39]. Further, we demonstrated that 5A3-antibody-treated mice (IS model) developed hepatic lesions and marked inflammation as early as 2 dpi, with severe hepatocellular degeneration and necrosis observed by day 4, recapitulating the severe hepatic injury seen in human CCHF cases [12]. In this study, we observed many ISGs, such as *Oasl1*, *Ifit3*, and *Gbp2*, that were strongly induced in both infections, with fold changes increasing progressively from 2 to 4 days post-infection (dpi), reflecting the activation of antiviral pathways. *Gbp2* exhibited higher fold changes in SFTSV infection compared to CCHFV, indicating virus-specific differences. Similarly, the strong upregulation of guanylate-binding proteins, *Gbp2* and *Gbp5,* in SFTSV infection is consistent with studies demonstrating that guanylate-binding proteins play a key role in restricting intracellular pathogens by various mechanisms, including the activation of inflammasomes [40].

Previous studies using NanoString analysis of CCHFV-infected mouse livers revealed significant upregulation of chemokines (*Ccl2*, *Cxcl1*, *Cxcl10*, *Cxcl9*) and cytokines (*Il1rn*, *Il1r2*, *Il17re*, *Il1a*, *Il1b*), alongside ISGs (*Ifit1*, *Ifit2*, *Ifnb1*, *Oasl1a*, *Oasl1*, *Stat1*, *Irf1*, *Irf7*), highlighting the critical role of IFN-I in modulating host immune responses during CCHF infection [12]. This study revealed a dramatic increase in many of these chemokines and cytokines with SFTSV and corroborates our previous findings with CCHFV infection in mice. Chemokines like *Cxcl9, Cxcl10*, *and Ccl2,* which recruit immune cells like macrophages and T cells, while interleukin-related genes such as *Il1r2* modulate inflammatory responses. *Il1r2*, a negative regulator of inflammation known for its anti-inflammatory effects by sequestering *Il1α* and *Il1β,* was upregulated significantly in both CCHFV and SFTSV-infected mice, indicating a host regulatory mechanism to reduce inflammation [41]. Notably, the monocyte chemokine MCP-1 (CCL2) was strongly associated with severe CCHF disease in humans [14], exhibiting a time-dependent increase and remaining persistently elevated during the later stages of disease in our data. Temporal dynamics revealed a progressive increase in the magnitude of gene expression changes from 2 dpi to 4 dpi, suggesting an escalating immune response as the infection advanced. These findings suggest that these markers could serve as prognostic indicators to predict disease severity and mortality with both viruses, providing valuable insights for clinical management and therapeutic development.

Further, we identified distinct gene expression patterns specific to CCHFV and SFTSV infections. Early induction of interferon genes was observed at 2 dpi, with persistently high expression during CCHFV infection. In contrast, these transcripts were suppressed in SFTSV infection, indicating a negative correlation between viral load and type I interferon responses in SFTSV [42]. Additionally, we identified genes exclusively altered during SFTSV infection, including *Ccr5*, *Cxcl12*, *Cd40lg*, *Il5ra*, and *Ptger4*, which may serve as potential biomarkers to distinguish CCHF from SFTS. Clinical studies have demonstrated that severe and fatal SFTSV cases are associated with downregulation of IFN-α and IFN-β, alongside high viremia and elevated proinflammatory cytokines such as IL-1β, IL-6, and TNF-α, which strongly correlate with disease severity [42,43]. Similarly, in CCHFV infection, elevated *Tnf-α* and *Il-6* levels are linked to severe and fatal outcomes in humans, and we observed marginal induction of both during later stages of disease in this study, indicating poor prognosis. Consistent with clinical findings, our previous study revealed upregulation of TNF superfamily genes during CCHFV infection [12]. Further supporting these observations, CCHFV (Afg09-2990) infected TNF-α receptor knockout mice treated with 5A3 antibody (IS model) exhibited reduced viral titers, delayed mean time-to-death, and decreased inflammatory cytokines and chemokines. This resulted in limited liver injury and improved survival outcomes compared to untreated controls [18]. These findings underscore the critical role of type I interferons and inflammatory cytokines in disease progression and highlight their importance as potential therapeutic targets.

SFTSV evades innate immune response using various mechanisms by reducing NK cell subsets, interfering with NF-κB signaling, regulating autophagy, and inhibiting the IFN signaling pathway [44]. Similarly, the downregulation of *Stat4* and *Irf4* observed in this study, particularly in SFTSV infection, is consistent with reports that SFTSV nonstructural proteins inhibit interferon production by targeting IRF3 and IRF7, which are key transcription factors in the interferon signaling cascade [15,44,45,46]. Furthermore, SFTSV NS inhibited JAK/STAT signaling by decreasing p-STAT1 level, suppressed Interferon-Stimulated Response Element (ISRE) activity, and downregulated the ISG expression to evade host immune surveillance [47]. This corroborates our observation that the key interferons were suppressed during SFTSV infection. The observed reduction of ISGs aligns with established immune evasion strategies employed by CCHF. For example, CCHFV suppresses type I interferon signaling by targeting components of the JAK-STAT pathway, such as *Stat1* and *Stat2*, through its viral ovarian tumor protease, which deubiquitinates and destabilizes these proteins, ultimately impairing ISG induction to suppress innate antiviral immunity [48]. Similarly, SFTSV has been reported to inhibit interferon production by interfering with key transcription factors like IRF3 and IRF7, further supporting the notion that both viruses actively manipulate host immune pathways to establish infection. These findings collectively highlight the interplay between host antiviral defense and viral immune evasion, emphasizing the need for further studies to identify targets that can enhance host immunity while resisting viral evasion strategies.

Toll-like receptors (TLRs) are essential components of the innate immune system, recognizing pathogen-associated molecular patterns (PAMPs) and initiating immune responses. In the context of SFTSV, monocytes, which are the primary target cells, exhibit significant alterations in TLR expression. TLR3 expression in monocytes decreases progressively with increasing disease severity in patients, from mild to severe and fatal cases, suggesting that TLR pathway impairment may be a critical mechanism for suppressing innate immune responses and facilitating immune evasion [13,42]. Similarly, in both CCHFV and SFTSV infections, TLRs play distinct roles in viral RNA sensing, influencing antiviral defense and disease pathogenesis. Polymorphisms in TLRs, including *TLR-3*, *-7*, *-8*, *-9*, and *-10*, have been associated with increased susceptibility, disease severity, and higher fatality rates, highlighting their importance in clinical outcomes [49,50,51,52]. Our findings revealed upregulation of *Tlr-2*, *-4*, *-6*, *-7*, and *-8*, alongside downregulation of *Tlr-9* in both infections, while *Tlr-3* and *-5* were uniquely upregulated in CCHFV (Supplemental Table S1). These results suggest that TLR polymorphisms and differential regulation of TLR pathways may alter host susceptibility and contribute to distinct immune responses in CCHFV and SFTSV infections. The observed downregulation of *Tlr3* in SFTSV-infected monocytes, particularly in severe and fatal cases, further supports the hypothesis that TLR pathway impairment is a key mechanism of immune suppression. Collectively, these findings underscore the potential of TLRs as therapeutic targets and biomarkers for disease severity, warranting further investigation into their roles in modulating host-pathogen interactions.

## 5. Conclusions

We utilized a well-established murine IS model to investigate the temporal transcriptional changes during SFTSV and CCHFV infections. Our findings revealed dynamic alterations in immune response pathways, including chemokines, cytokines, ISGs, and TLRs, alongside temporal changes in target genes that could serve as potential biomarkers for monitoring disease progression and severity. Notably, we demonstrated that the two viruses differ in the severity of immune responses, with CCHFV displaying larger magnitude responses than SFTSV, reflecting distinct pathogenesis. Several markers identified in this study have been validated in clinical studies, further supporting their potential as reliable biomarkers for evaluating the efficacy of vaccines and therapeutics in preclinical research. Further, we identified both shared gene expression patterns and unique genes that distinguish CCHFV from SFTSV, providing critical insights into the molecular mechanisms underlying these infections. However, the study is limited by the use of a murine model, which may not fully replicate human immune responses, and does not include changes at the translational level. Further, due to the nature of CCHF and SFTS disease, changes in the blood cell populations could account for some of the gene expression changes that we see here. Further studies to include blood cell population analysis can be performed to control for this. Additionally, the absence of clinical data highlights the need for further validation as well as studies in human populations. Despite these limitations, our study uncovers several potential therapeutic targets and biomarkers that could be leveraged to assess disease progression, severity, and the efficacy of medical countermeasures. These findings represent a significant advancement in understanding the pathogenesis of CCHFV and SFTSV and offer valuable tools for improving clinical outcomes.

## Figures and Tables

**Table 1:** Unique genes affected during CCHFV and SFTSV infection.

Gene Symbol	CCHFV						Gene Symbol	SFTSV					
	2 dpi		3 dpi		4 dpi			2 dpi		3 dpi		4 dpi	
	Fold Change	Adj. *p*-Value	Fold Change	Adj. *p*-Value	Fold Change	Adj. *p*-Value		Fold Change	Adj. *p*-Value	Fold Change	Adj. *p*-Value	Fold Change	Adj. *p*-Value
*Ifnb1*	13.0	0.009	334.4	0.000	221.3	0.005	*Ccr5*	6.8	0.000	7.7	0.000	8.7	0.000
*Ifna4*	12.3	0.012	305.4	0.000	129.4	0.001	*Cxcl12*	2.5	0.188	5.3	0.033	6.2	0.020
*Ifna2*	6.4	0.048	87.8	0.000	29.8	0.028	*Vwf*	1.4	0.331	4.0	0.003	3.7	0.007
*Ifnl2/3*	4.6	0.123	71.4	0.001	29.7	0.009	*Samhd1*	3.1	0.000	3.8	0.000	3.3	0.000
*Ifna*	5.3	0.029	67.1	0.000	17.7	0.005	*Casp1*	3.2	0.000	3.4	0.000	2.9	0.000
*Defa1*	3.6	0.184	57.0	0.000	10.5	0.044	*Pak1*	2.5	0.015	3.2	0.005	3.6	0.002
*Ddah2*	3.8	0.126	33.2	0.001	14.3	0.016	*Psmb9*	2.8	0.000	2.9	0.000	2.6	0.000
*Il17rc*	3.8	0.141	31.6	0.002	16.5	0.014	*Psmb10*	2.3	0.000	2.8	0.000	3.0	0.000
*Ccl22*	5.3	0.034	17.1	0.001	7.1	0.023	*Tap2*	2.1	0.003	2.5	0.000	2.4	0.001
*Il17rb*	3.8	0.170	22.8	0.004	11.5	0.032	*Tyrobp*	2.1	0.000	2.5	0.000	2.6	0.000
*Cxcl3*	2.8	0.206	16.0	0.007	18.0	0.006	*Stat3*	1.6	0.011	2.0	0.002	2.4	0.000
*Il11*	1.0	1.000	14.2	0.014	10.5	0.037	*Eif2ak3*	−2.6	0.007	−2.6	0.007	−2.7	0.006
*Il17b*	2.2	0.398	11.8	0.020	10.5	0.032	*Ptger4*	−9.7	0.001	−3.5	0.008	−4.4	0.005
*Cxcl14*	1.6	0.586	8.2	0.030	8.8	0.029	*Atm*	−2.6	0.010	−4.2	0.002	−4.9	0.001
*Fasl*	2.2	0.253	8.2	0.009	8.7	0.008	*Sigirr*	−5.8	0.003	−4.7	0.009	−2.0	0.079
*Sting*	−1.5	0.012	−2.2	0.001	−2.5	0.000	*Il5ra*	−4.3	0.004	−5.6	0.001	−3.2	0.010
*Ctla4*	7.0	0.009	5.5	0.018	6.6	0.013	*Cd40lg*	−4.6	0.003	−6.2	0.001	−8.7	0.000
*Ccr2*	−2.5	0.001	−4.3	0.000	−5.0	0.000	*Bcl2*	−3.1	0.009	−8.1	0.000	−4.1	0.005
*Ccl21a/b/c/d*	4.0	0.122	26.2	0.001	10.7	0.026	*Pdcd1lg2*	−6.7	0.000	−6.4	0.000	−4.2	0.001

Values represent fold changes in relative expression compared to uninfected mice. Statistical significance of the fold changes is indicated by adjusted *p*-values (Adj. *p*-values). dpi: days post-infection.

**Table 2:** Temporal changes in interferon stimulatory genes during CCHFV and SFTSV infection.

Gene Symbol			CCHFV						SFTSV			
	2 dpi		3 dpi		4 dpi		2 dpi		3 dpi		4 dpi	
	Fold Change	Adj. *p*-Value	Fold Change	Adj. *p*-Value	Fold Change	Adj. *p*-Value	Fold Change	Adj. *p*-Value	Fold Change	Adj. *p*-Value	Fold Change	Adj. *p*-Value
*Oasl1*	42.6	0.000	167.4	0.000	153.3	0.000	32.4	0.000	31.6	0.000	55.8	0.000
*Oas2*	17.5	0.000	27.1	0.000	23.3	0.000	23.4	0.000	32.0	0.000	29.7	0.000
*Oas1a*	9.2	0.000	24.4	0.000	32.2	0.000	15.1	0.000	20.8	0.000	28.0	0.000
*Oas3*	2.4	0.014	5.6	0.000	4.6	0.002	3.6	0.001	3.4	0.001	4.5	0.000
*Ifit3*	21.6	0.000	117.0	0.000	132.5	0.000	50.8	0.000	36.9	0.000	47.3	0.000
*Ifit1*	7.5	0.000	29.9	0.000	28.1	0.000	6.8	0.000	9.1	0.000	11.9	0.000
*Ifit2*	1.8	0.052	5.6	0.002	8.8	0.000	2.0	0.002	1.6	0.021	2.1	0.001
*Ifitm1*	5.9	0.004	3.0	0.018	13.2	0.000	21.9	0.000	24.6	0.000	36.5	0.000
*Ifitm2*	3.5	0.000	2.7	0.000	4.4	0.000	4.5	0.000	6.0	0.000	7.6	0.000
*Ifitm3*	3.4	0.000	6.3	0.000	8.3	0.000	7.2	0.000	10.6	0.000	15.3	0.000
*Ifnlr1*	9.8	0.005	27.2	0.000	17.8	0.002	5.6	0.034	17.6	0.002	18.2	0.002
*Ifnz*	6.7	0.015	72.6	0.000	33.7	0.001	1.7	0.232	3.9	0.013	3.6	0.019
*Ifna1/5/6/12/13/14*	5.3	0.029	67.1	0.000	17.7	0.005	1.8	0.294	2.5	0.101	2.4	0.125
*Gbp2*	41.6	0.000	19.9	0.000	24.9	0.000	116.4	0.000	101.1	0.000	65.7	0.000
*Gbp5*	15.6	0.000	11.2	0.000	9.2	0.000	27.0	0.000	32.0	0.000	17.9	0.000
*Gbp3*	3.8	0.000	3.6	0.000	5.3	0.000	7.0	0.000	7.6	0.000	4.8	0.000
*Socs3*	10.7	0.000	4.4	0.000	7.7	0.000	9.8	0.000	12.5	0.000	11.9	0.000
*Socs1*	8.2	0.018	12.6	0.006	11.7	0.009	9.0	0.007	21.2	0.000	11.8	0.004
*Ifi44*	3.8	0.087	147.2	0.000	213.8	0.000	1.4	0.736	18.2	0.022	90.4	0.000
*Mx1*	3.4	0.134	47.6	0.002	90.5	0.000	1.2	0.851	3.2	0.245	8.4	0.026
*Ifih1(mda5)*	2.9	0.000	9.8	0.000	11.1	0.000	4.1	0.000	4.0	0.000	5.9	0.000
*Bst2*	2.4	0.010	10.3	0.000	9.0	0.001	4.1	0.004	5.3	0.002	11.9	0.000
*Eif2ak2*	2.6	0.001	4.5	0.000	3.0	0.001	3.1	0.000	3.3	0.000	3.1	0.000
*Stat2*	2.4	0.005	3.1	0.001	2.4	0.007	3.5	0.000	4.1	0.000	4.3	0.000
*Stat4*	−3.8	0.042	1.2	0.457	−7.1	0.006	−10.8	0.000	−10.8	0.000	−4.2	0.002
*Trim6*	−1.2	0.744	−2.3	0.198	−1.0	0.955	−2.3	0.214	−3.9	0.061	−1.0	0.959
*Trim26*	−2.1	0.000	−1.2	0.282	−1.9	0.008	−1.7	0.026	−2.0	0.004	−1.7	0.026
*Irf7*	1.5	0.058	6.0	0.000	5.2	0.002	2.1	0.148	2.0	0.173	7.0	0.000
*Irf4*	−3.0	0.015	−4.0	0.004	−3.3	0.014	−6.3	0.004	−11.8	0.000	−7.0	0.003
*Rsad2*	−2.7	0.080	3.0	0.000	−2.8	0.098	−1.0	0.932	−2.0	0.231	−3.3	0.032
*Trim5*	−1.7	0.088	−1.7	0.081	−3.0	0.001	−2.5	0.000	−2.2	0.001	−2.1	0.001

Values represent fold changes in relative expression compared to uninfected mice. Statistical significance of the fold changes is indicated by adjusted *p*-values (Adj. *p*-values). dpi: days post-infection.

**Table 3:** Highly upregulated chemokines and cytokines during CCHFV and SFTSV infection.

Gene Symbol	CCHFV						Gene Symbol	SFTSV					
	2 dpi		3 dpi		4 dpi			2 dpi		3 dpi		4 dpi	
	Fold Change	Adj. *p*-Value	Fold Change	Adj. *p*-Value	Fold Change	Adj. *p*-Value		Fold Change	Adj. *p*-Value	Fold Change	Adj. *p*-Value	Fold Change	Adj. *p*-Value
*Cxcl9*	297.6	0.000	661.8	0.000	1208.8	0.000	*Cxcl9*	387.8	0.000	1000.4	0.000	409.4	0.000
*Cxcl10*	280.6	0.000	1004.4	0.000	2184.7	0.000	*Cxcl10*	142.4	0.000	373.6	0.000	101.2	0.000
*Ccl2*	84.5	0.000	1099.8	0.000	3108.3	0.000	*Cxcl1*	99.3	0.000	191.9	0.000	112.7	0.000
*Cxcl1*	83.6	0.000	158.2	0.000	151.2	0.000	*Ccl2*	20.6	0.000	73.2	0.000	79.7	0.000
*Cxcl2*	57.8	0.000	45.0	0.000	75.2	0.000	*Ccl12*	15.2	0.005	1.8	0.497	7.6	0.043
*Ccl12*	34.2	0.001	92.8	0.002	183.2	0.000	*Ccrl2*	12.8	0.000	22.1	0.000	19.9	0.000
*Ccrl2*	31.0	0.000	42.1	0.000	34.7	0.000	*Cxcr2*	8.4	0.000	9.0	0.000	8.2	0.000
*Ccl3*	28.0	0.002	93.4	0.002	107.4	0.002	*Ccr5*	6.8	0.000	7.7	0.000	8.7	0.000
*Cxcl11*	26.8	0.005	149.2	0.007	311.9	0.002	*Cxcl2*	1.80	0.502	36.40	0.004	59.00	0.001
*Ccl7*	23.2	0.003	201.1	0.002	310.5	0.000	*Ccl7*	5.8	0.038	44.2	0.001	40.4	0.001
*Cxcr2*	5.9	0.000	2.5	0.023	3.9	0.006	Ccr1	6.4	0.000	8.0	0.000	9.8	0.000
*Il1r2*	63.2	0.000	90.0	0.000	283.1	0.000	*Il1r2*	92.8	0.000	220.3	0.000	331.8	0.000
*Il18bp*	14.8	0.000	13.6	0.000	12.1	0.000	*Il18bp*	36.5	0.000	43.7	0.000	46.2	0.000
*Il1rn*	10.8	0.000	19.0	0.000	34.4	0.000	*Il15ra*	24.8	0.000	41.6	0.000	53.2	0.000
*Il1f9*	10.8	0.000	7.3	0.000	8.6	0.000	*Il1f9*	10.5	0.000	10.6	0.000	8.0	0.000
*Il15ra*	10.2	0.004	35.2	0.000	39.3	0.000	*Il1rn*	10.4	0.000	8.3	0.000	8.5	0.000
*Il6*	9.6	0.012	74.2	0.000	39.2	0.002	*Il1r1*	6.3	0.001	8.5	0.000	5.5	0.001
*Il1a*	6.8	0.030	47.0	0.001	42.3	0.340	*Il1rap*	5.8	0.000	6.3	0.000	5.8	0.000
*Tnf*	5.8	0.002	9.1	0.000	5.0	0.002	*Ifnlr1*	5.6	0.034	17.6	0.002	18.2	0.002
*Il1rap*	5.7	0.000	2.8	0.002	4.1	0.003	*Tnf*	1.9	0.178	6.0	0.001	3.2	0.044
*Il34*	4.6	0.105	39.2	0.001	16.3	0.001	*Il6*	1.0	1.000	46.40	0.002	19.00	0.019

Values indicate fold changes and adjusted (adj.) *p*-values compared to uninfected mice.

## Data Availability

The raw data supporting the conclusions of this article will be made available by the authors on request.

## Supplementary Materials

The following supporting information can be downloaded at: https://www.mdpi.com/article/10.3390/v18050504/s1, Table S1. Fold changes in Toll-like receptors during CCHFV and SFTSV infection in mice. Table S2. Comparison of the top 50 highly upregulated and downregulated genes during CCHFV and SFTSV infection. Table S3. Top 15 upregulated and downregulated pathways identified by “REACTOME” during CCHFV infection in mice. Table S4. Top 15 upregulated and downregulated pathways identified by “REACTOME” during SFTSV infection in mice. Figure S1. Venn diagrams illustrate the overlap and divergence in gene expression changes between CCHFV and SFTSV on days 2, 3, and 4 post-infections.

## Abbreviations

The following abbreviations are used in this manuscript:

CCHFV	Crimean–Congo hemorrhagic fever virus
SFTSV	Severe Fever with Thrombocytopenia Syndrome Virus
IFN	Interferon
ISG	Interferon Stimulated Genes
TNF	Tumor Necrosis Factor
IFNAR	Interferon Alpha Receptor
IS	Immunosuppression
(A)BSL-4	(Animal) Biosafety level 4
USAMRIID	United States Army Medical Research Institute of Infectious Diseases
IACUC	Institutional Animal Care and Use Committee
RT-qPCR	Reverse Transcriptase quantitative Polymerase Chain Reaction
RNA	Ribonucleic Acid
GO	Gene Ontology
GCE/rxn	Genomic Copy Equivalents per reaction
Dpi	Days post infection
DEG	Differentially Expressed Gene
PCA	Principal Component Analysis
TLR	Toll Like Receptor
PAMP	Pattern Associated Molecular Pattern
