# Supplementary material for: Dynamic Changes in Host Immune Response During Crimean–Congo Hemorrhagic Fever and Severe Fever with Thrombocytopenia Syndrome in Mice
Source: Viruses. 2026 Apr 28;18(5):504. doi: 10.3390/v18050504 (PMC13211718; doi:10.3390/v18050504)
Supplement: Supplementary file 1 [file viruses-18-00504-s001.zip › Supplementary tables_1-4.pdf]

Supplemental table S1. Fold changes in Toll-like receptors during CCHFV and SFTSV infection mice.

Gene symbol	CCHFV						SFTSV					
	2 dpi		3 dpi		4 dpi		2 dpi		3 dpi		4 dpi	
	Fold change	Adj. p-value	Fold change	Adj. p-value	Fold change	Adj. p-value	Fold change	Adj. p-value	Fold change	Adj. p-value	Fold change	Adj. p-value
Tlr2	11.2	0.000	8.5	0.000	7.4	0.000	6.6	0.000	9.2	0.000	8.0	0.000
Tlr4	3.0	0.001	2.3	0.002	4.2	0.000	4.4	0.000	5.4	0.000	5.3	0.000
Tlr5	3.2	0.191	23.6	0.002	9.8	0.033	NA					
Tlr6	2.1	0.018	3.0	0.002	2.8	0.004	3.4	0.001	3.5	0.001	3.6	0.001
Tlr7	1.1	0.850	6.7	0.005	7.3	0.005	1.5	0.320	3.9	0.019	6.4	0.001
Tlr8	3.1	0.041	2.3	0.103	5.1	0.004	3.4	0.010	7.3	0.002	13.2	0.000
Tlr3	-1.1	0.775	10.7	0.002	7.5	0.017	NA					
Tlr9	-2.0	0.019	-1.4	0.145	-2.5	0.002	-3.3	0.005	-2.5	0.025	-3.3	0.009
Tlr1	-2.0	0.005	-2.5	0.001	.2.0	0.002	NA					

NA- not detected/no changes were observed compared to uninfected mice.

Supplemental table S2. Comparison of top 50 highly upregulated and downregulated genes during CCHFV and SFTSV infection.

CCHFV						SFTSV					
2 dpi		3 dpi		4 dpi		2 dpi		3 dpi		4 dpi	
Symbol	log2 fold change	Symbol	log2 fold change	Symbol	log2 fold change	Symbol	log2 fold change	Symbol	log2 fold change	Symbol	log2 fold change
Cxcl9	8.2	Ccl2	10.1	Ccl2	11.6	*Cxcl9*	8.6	Cxcl9	10.0	Cxcl9	8.7
Cxcl10	8.1	Cxcl10	10.0	Cxcl10	11.1	*Cxcl10*	7.2	Cxcl10	8.6	Il1r2	8.4
Ccl2	6.4	Cxcl9	9.4	Cxcl9	10.2	*Gbp2*	6.9	Il1r2	7.8	Lcn2	7.5
Cxcl1	6.4	Ifnb1	8.4	Cxcl11	8.3	*Cxcl1*	6.6	Cxcl1	7.6	Ltf	7.1
Il1r2	6.0	Ifna4	8.3	Ccl7	8.3	*Il1r2*	6.5	Lcn2	7.4	Nos2	6.9
Cxcl2	5.9	Ccl7	7.7	Il1r2	8.2	*Lcn2*	6.1	Cd8b1	-6.9	Cxcl1	6.8
Lcn2	5.8	Oasl1	7.4	Ifnb1	7.8	*Ifit3*	5.7	Lef1	-6.7	Cxcl10	6.7
Ltf	5.8	Cxcl1	7.3	Ifi44	7.7	*Cx3cr1*	-5.4	Gbp2	6.7	Ifi44	6.5
Cd14	5.5	Cxcl11	7.2	Ccl12	7.5	*Ccr6*	-5.3	Ccr6	-6.4	Cd14	6.4
Oasl1	5.4	Ifi44	7.2	Oasl1	7.3	*Fcgr1*	5.2	Cd4	-6.3	Ccl2	6.3
Gbp2	5.4	Ifit3	6.9	Cxcl1	7.2	*Il18bp*	5.2	Ccl2	6.2	Mapk13	6.0
Ace	-5.1	Lcn2	6.6	Lcn2	7.1	*Mgam*	5.2	Nos2	6.1	Gbp2	6.0
Ccl12	5.1	Ccl3	6.5	Ifit3	7.1	*Hdc*	5.1	Lrg1	6.0	Lrg1	6.0
Ccrl2	5.0	Ccl12	6.5	Ifna4	7.0	*Oasl1*	5.0	Ltf	5.9	Ccr9	-6.0
Ccl3	4.8	Il1r2	6.5	Ltf	6.9	*Crp*	5.0	Trat1	-5.7	Cxcl2	5.9
Cxcl11	4.7	Ifna2	6.5	Ccl3	6.8	*Hspb1*	5.0	Mapk13	5.6	Oasl1	5.8
Crp	4.6	Hspb1	6.4	Crp	6.7	*Mapk13*	4.9	Cd14	5.6	Il15ra	5.7
Ccl7	4.5	Il6	6.2	Mx1	6.5	*Gbp5*	4.8	Il6	5.5	Ccr6	-5.7
Hdc	4.4	Ifnz	6.2	Cxcl2	6.2	*Lrg1*	4.7	Ccl7	5.5	Fpr2	5.6
Ifit3	4.4	IFNL2/3	6.2	Cd14	5.9	*Ltf*	4.7	Il18bp	5.5	Fcgr1	5.6
Fpr1	4.4	IFNA	6.1	Hspb1	5.4	*Il15ra*	4.6	Cd3g	-5.4	Ifit3	5.6
Lrg1	4.3	Ltf	5.9	Gzmb	5.4	*Fpr2*	4.6	Il15ra	5.4	Cd8b1	-5.5
Fpr2	4.2	NA	5.8	Il1a	5.4	*Oas2*	4.6	Fcgr1	5.4	Il18bp	5.5
Nos2	4.2	Mx1	5.6	Nos2	5.3	*Ifitm1*	4.5	Tcf7	-5.4	Hamp	5.5
Oas2	4.1	Il1a	5.6	Cd27	-5.3	*Ccl2*	4.4	Crp	5.4	Igfbp7	5.4
Mgam	4.1	Crp	5.5	Il15ra	5.3	*Ccr3*	-4.3	Mgam	5.3	Mgam	5.4
Fcgr1	4.0	Plin4	5.5	Il6	5.3	*Tgfb2*	-4.2	Hdc	5.2	Elane	5.4
Cx3cr1	-4.0	Igfbp7	5.5	Il10	5.2	*Fpr1*	4.2	Sh2d1a	-5.2	Hspb1	5.4
Mefv	4.0	Cxcl2	5.5	Ccrl2	5.1	*Ace*	-4.1	Ifit3	5.2	Ccl7	5.3
Gbp5	4.0	Ccl1	5.5	Il1rn	5.1	*Aif1*	4.1	Cxcl2	5.2	Hdc	5.3
Mapk13	3.9	Nos2	5.4	Ifnz	5.1	*Igfbp7*	4.1	Fpr2	5.2	Ifitm1	5.2
Il18bp	3.9	Il9	5.4	Oas1a	5.0	*Cd14*	3.9	Hamp	5.1	Cd8a	-5.1
Bcl3	3.8	Ccrl2	5.4	Mapk13	5.0	*Ccl12*	3.9	Cd96	-5.0	Il1a	5.0
Igfbp7	3.7	Il34	5.3	Fpr2	5.0	*Oas1a*	3.9	Gbp5	5.0	Oas2	4.9
Ifnb1	3.7	Cxcl15	5.2	Cd8a	-4.9	*C3*	3.9	Oas2	5.0	Ccl3	4.9
Ebi3	3.6	Nod2	5.2	Ifna2	4.9	*Fcgr4*	3.8	Txk	-5.0	Oas1a	4.8
Ifna4	3.6	Il15ra	5.1	IFNL2/3	4.9	*Mefv*	3.7	Oasl1	5.0	Lef1	-4.8
Osm	3.5	Vcam1	5.1	Ccl4	4.9	*Ccrl2*	3.7	C3	5.0	Fpr1	4.8
Tlr2	3.5	Ddah2	5.1	Plscr2	4.8	*Ebi3*	3.5	Cd3e	-4.9	C3	4.8
Il1rn	3.4	Cd14	5.0	Trat1	-4.8	*Stat4*	-3.4	Cd8a	-4.9	Tcf7	-4.7
Il1f9	3.4	Cd27	-5.0	Ifit1	4.8	*Zbp1*	3.4	Hspb1	4.9	Crp	4.7
Socs3	3.4	Ccl4	5.0	Lrg1	4.8	*Il1f9*	3.4	Cd3d	-4.9	Cd4	-4.7
Il15ra	3.4	Il17rc	5.0	Hmox1	4.7	*Il1rn*	3.4	Cd27	-4.9	Mafb	4.6
Ifnlr1	3.3	Acvr1	5.0	Cd8b1	-4.7	*Hamp*	3.3	Igfbp7	4.9	Hc	4.6
Il6	3.3	KIR3DL1/2	4.9	Fpr1	4.7	*Socs3*	3.3	Lck	-4.8	Il10	4.5
Hamp	3.2	Neo1	4.9	Gbp2	4.6	*Nod2*	3.3	Itk	-4.7	Anpep	4.5
Il1b	3.2	Il23r	4.9	Igfbp7	4.6	*Ptger4*	-3.3	Ifitm1	4.6	Plg	4.4
Hspb1	3.2	Ifit1	4.9	Oas2	4.5	*Nampt*	3.2	Fpr1	4.6	Ebi3	4.4
Oas1a	3.2	Ms4a7	4.9	Il23a	4.5	*Socs1*	3.2	Il1a	4.6	Fcgr4	4.4
Nod2	3.2	Ager	4.8	Cd4	-4.5	*Plscr1*	3.1	Ccrl2	4.5	Ccrl2	4.3

Values are log2 fold changes of genes altered by more than two-fold with adjusted p-value <0.05.

Supplemental table S3. Top 15 upregulated and downregulated pathways identified by 'REACTOME' during CCHFV infection in mice.

2 dpi				3 dpi				4 dpi			
**Pathways**	**Adj. p-value**	**nGenes**	**Fold enriched**	**Pathways**	**Adj. p-value**	**nGenes**	**Fold enriched**	**Pathways**	**Adj. p-value**	**nGenes**	**Fold enriched**
**Upregulated pathways**											
Immune System	1.47e-33	83	3.8	Immune System	6.26e-48	136	3.4	Immune System	1.83e-48	122	3.7
Cytokine Signaling in Immune system	1.36e-19	36	7.2	Cytokine Signaling in Immune system	4.95e-41	69	7.6	Innate Immune System	1.99e-26	78	3.8
Signaling by Interleukins	1.27e-16	28	8.3	Signaling by Interleukins	1.30e-31	51	8.4	Cytokine Signaling in Immune system	4.47e-25	49	6.5
Innate Immune System	1.87e-14	48	3.5	Chemokine receptors bind chemokines	2.08e-30	28	22.8	Signaling by Interleukins	2.47e-21	38	7.5
Chemokine receptors bind chemokines	1.30e-10	12	17.6	Peptide ligand-binding receptors	1.05e-22	37	8.5	Chemokine receptors bind chemokines	5.77e-18	19	18.6
Peptide ligand-binding receptors	1.05e-08	17	7.1	Innate Immune System	5.34e-16	71	2.9	Toll-like Receptor Cascades	9.57e-16	25	9.1
Toll-like Receptor Cascades	1.34e-08	15	8.2	Class A/1 Rhodopsin-like receptors	7.23e-16	38	5.3	Peptide ligand-binding receptors	7.47e-14	26	7.2
Toll Like Receptor 4 TLR4 Cascade	9.08e-07	12	8.1	GPCR ligand binding	1.94e-12	38	4.1	Toll Like Receptor 4 TLR4 Cascade	1.92e-12	20	9.0
Neutrophil degranulation	1.74e-06	24	3.5	G alpha signaling events	1.87e-10	29	4.6	Neutrophil degranulation	1.92e-12	40	3.9
Interferon alpha/beta signaling	1.74e-06	7	18.8	Toll-like Receptor Cascades	3.78e-10	21	6.4	MyD88-independent TLR4 cascade	1.24e-10	17	9.0
Interleukin-1 signaling	1.74e-06	11	8.5	Interleukin-1 family signaling	4.75e-10	19	7.1	TRIFTICAM1-mediated TLR4 signaling	1.24e-10	17	9.0
Interferon Signaling	5.48e-06	9	10.1	Toll Like Receptor 4 TLR4 Cascade	3.91e-09	18	6.8	Toll Like Receptor 7/8 TLR7/8 Cascade	2.53e-10	16	9.5
Interleukin-1 family signaling	5.48e-06	11	7.5	Toll Like Receptor 7/8 TLR7/8 Cascade	3.99e-09	16	7.9	Class A/1 Rhodopsin-like receptors	1.14e-09	27	4.5
Class A/1 Rhodopsin-like receptors	8.54e-06	17	4.2	Signaling by GPCR	7.18e-09	41	2.9	MyD88 dependent cascade	2.24e-09	15	9.0
MyD88-independent TLR4 cascade	8.54e-06	10	8	MyD88 dependent cascade	2.93e-08	15	7.5	Toll Like Receptor 9 TLR9 Cascade	4.65e-09	15	8.5
**Downregulated pathways**											
Toll Like Receptor 7/8 TLR7/8 Cascade	2.82e-05	9	8	Immune System	1.55e-26	72	3.6	Immune System	2.93e-26	67	3.8
Toll Like Receptor 9 TLR9 Cascade	3.86e-05	9	7.6	Adaptive Immune System	1.73e-15	41	4.5	Adaptive Immune System	1.60e-19	43	5.3
Immune System	1.21e-04	17	3.2	Cytokine Signaling in Immune system	4.92e-13	28	6.1	TCR signaling	1.14e-17	20	17.4
Chemokine receptors bind chemokines	1.25e-03	4	24.3	Generation of second messenger molecules	1.10e-12	11	32.5	Downstream TCR signaling	1.44e-13	16	16.5
Signaling by the B Cell Receptor BCR	1.49e-03	6	9.2	TCR signaling	9.54e-12	16	12.4	Generation of second messenger molecules	3.17e-13	11	36.4
Adaptive Immune System	1.87e-03	10	4.2	Signaling by Interleukins	1.30e-10	21	6.9	Translocation of ZAP-70 to Immunological synapse	5.88e-12	9	47.0
DAP12 signaling	2.12e-03	3	34.6	Innate Immune System	1.55e-10	40	3.2	Costimulation by the CD28 family	7.55e-12	13	19.0
DAP12 interactions	5.02e-03	3	24.5	Phosphorylation of CD3 and TCR zeta chains	3.08e-09	8	32.2	Phosphorylation of CD3 and TCR zeta chains	2.32e-11	9	40.6
Antigen activates B Cell Receptor	1.24e-02	4	9.8	Costimulation by the CD28 family	9.63e-09	11	14.3	Innate Immune System	2.95e-10	37	3.3
Translocation of ZAP-70 to Immunological synapse	2.28e-02	2	35.2	DAP12 signaling	3.10e-08	8	24.4	PD-1 signaling	1.95e-09	8	34.5
Phosphorylation of CD3 and TCR zeta chains	2.68e-02	2	30.4	Translocation of ZAP-70 to Immunological synapse	3.42e-08	7	32.6	Cytokine Signaling in Immune system	2.76e-09	22	5.4
PD-1 signaling	2.68e-02	2	29.1	PD-1 signaling	1.47e-07	7	26.9	Signaling by Interleukins	4.64e-07	16	5.9
Peptide ligand-binding receptors	2.86e-02	4	6.9	Downstream TCR signaling	2.95e-07	11	10.1	Toll-like Receptor Cascades	1.03e-06	12	8.1
Downstream TCR signaling	2.94e-02	3	10.5	DAP12 interactions	4.40e-07	8	17.3	Signaling by the B Cell Receptor BCR	9.45e-06	13	5.9
The AIM2 inflammasome	2.94e-02	1	334.5	Interleukin-2 signaling	5.91e-07	5	49.2	Toll Like Receptor 9 TLR9 Cascade	1.60e-05	9	9.4

Pathways are ranked based on their significance (Adj. P-value)

Supplemental table S4. Top 15 upregulated and downregulated pathways identified by 'Reactome' during SFTSV infection in mice.

2 dpi				3 dpi				4 dpi			
**Pathways**	**Adj. p-value**	**nGenes**	**Fold enriched**	**Pathways**	**Adj. p-value**	**nGenes**	**Fold enriched**	**Pathways**	**Adj. p-value**	**nGenes**	**Fold enriched**
**Upregulated pathways**											
Immune System	7.40e-31	81	3.7	Immune System	9.13e-44	121	3.5	Immune System	5.73e-52	139	3.6
Innate Immune System	5.65e-17	52	3.8	Innate Immune System	1.10e-28	83	3.9	Innate Immune System	3.11e-36	98	4.1
Signaling by Interleukins	2.72e-14	26	7.7	Cytokine Signaling in Immune system	3.01e-19	44	5.6	Neutrophil degranulation	3.35e-19	54	4.4
Cytokine Signaling in Immune system	9.46e-14	30	5.9	Signaling by Interleukins	1.89e-17	35	6.6	Cytokine Signaling in Immune system	9.03e-19	46	5.1
Toll-like Receptor Cascades	2.76e-08	15	8.2	Toll-like Receptor Cascades	4.06e-16	26	9.1	Signaling by Interleukins	1.50e-17	37	6.2
Neutrophil degranulation	4.12e-08	27	3.9	Neutrophil degranulation	1.57e-13	43	4.0	Toll-like Receptor Cascades	7.18e-16	27	8.3
Nucleotide-binding domain leucine rich repeat containing receptor NLR signaling pathways	2.39e-07	9	15.6	Toll Like Receptor 4 TLR4 Cascade	8.47e-11	19	8.2	Toll Like Receptor 4 TLR4 Cascade	7.97e-12	21	8.0
Peptide ligand-binding receptors	8.17e-07	15	6.2	Chemokine receptors bind chemokines	1.80e-09	13	12.1	Peptide ligand-binding receptors	3.50e-10	24	5.6
Chemokine receptors bind chemokines	9.77e-07	9	13.1	Peptide ligand-binding receptors	8.52e-09	21	5.6	MyD88-independent TLR4 cascade	3.08e-09	17	7.6
Toll Like Receptor 4 TLR4 Cascade	9.14e-06	11	7.4	Toll Like Receptor 7/8 TLR7/8 Cascade	9.97e-09	15	8.5	TRIFTICAM1-mediated TLR4 signaling	3.08e-09	17	7.6
Interleukin-1 family signaling	9.14e-06	11	7.4	MyD88-independent TLR4 cascade	3.81e-08	15	7.6	Interleukin-1 family signaling	4.72e-09	18	6.9
Class A/1 Rhodopsin-like receptors	1.61e-05	17	4.2	TRIFTICAM1-mediated TLR4 signaling	3.81e-08	15	7.6	Toll Like Receptor 7/8 TLR7/8 Cascade	4.89e-09	16	8.0
Interleukin-1 signaling	1.94e-05	10	7.7	Interleukin-1 family signaling	4.27e-08	16	6.9	Chemokine receptors bind chemokines	5.50e-09	13	10.7
TRAF6 mediated induction of NFkB and MAP kinases upon TLR7/8 or 9 activation	4.41e-05	9	8.1	TRAF6 mediated induction of NFkB and MAP kinases upon TLR7/8 or 9 activation	5.60e-08	14	8.1	MyD88 dependent cascade initiated on endosome	3.61e-08	15	7.6
MyD88 dependent cascade initiated on endosome	4.52e-05	9	8.0	MyD88 dependent cascade initiated on endosome	6.09e-08	14	8.0	Toll Like Receptor 9 TLR9 Cascade	7.36e-08	15	7.2
**Downregulated pathways**											
Immune System	1.24e-11	41	3.2	Immune System	2.98e-20	60	3.4	Immune System	4.10e-20	54	3.7
Adaptive Immune System	2.66e-09	26	4.4	Adaptive Immune System	9.39e-13	35	4.4	Adaptive Immune System	1.85e-16	36	5.4
Chemokine receptors bind chemokines	5.63e-07	8	19.9	Generation of second messenger molecules	1.75e-11	10	33.8	Generation of second messenger molecules	3.95e-12	10	40.1
TCR signaling	6.95e-07	10	12.0	Costimulation by the CD28 family	1.82e-10	12	17.9	Costimulation by the CD28 family	2.91e-11	12	21.3
JNK c-Jun kinases phosphorylation and activation mediated by activated human TAK1	1.01e-06	6	34.2	Cytokine Signaling in Immune system	2.55e-10	23	5.7	TCR signaling	3.65e-11	14	14.8
Cytokine Signaling in Immune system	1.29e-06	16	5.4	TCR signaling	2.55e-10	14	12.5	Translocation of ZAP-70 to Immunological synapse	9.69e-11	8	50.7
Downstream TCR signaling	1.31e-06	9	12.8	Translocation of ZAP-70 to Immunological synapse	2.55e-10	8	42.7	Cytokine Signaling in Immune system	4.59e-10	21	6.2
Interleukin-17 signaling	8.79e-06	7	16.0	Signaling by Interleukins	5.35e-10	19	7.1	PD-1 signaling	4.59e-10	8	41.9
MAP kinase activation	8.79e-06	7	16.0	PD-1 signaling	1.25e-09	8	35.3	Downstream TCR signaling	1.70e-08	11	13.8
Toll Like Receptor 9 TLR9 Cascade	1.27e-05	8	11.5	Phosphorylation of CD3 and TCR zeta chains	4.30e-08	7	32.3	Phosphorylation of CD3 and TCR zeta chains	1.70e-08	7	38.3
Signaling by the B Cell Receptor BCR	1.27e-05	11	6.9	Downstream TCR signaling	7.32e-08	11	11.6	Signaling by Interleukins	2.16e-07	15	6.7
Costimulation by the CD28 family	1.35e-05	7	14.1	Chemokine receptors bind chemokines	1.80e-06	8	14.8	Signaling by the B Cell Receptor BCR	8.42e-06	12	6.7
PD-1 signaling	1.35e-05	5	29.7	DAP12 signaling	9.41e-06	6	21.0	Immunoregulatory interactions between a Lymphoid and a non-Lymphoid cell	9.75e-05	11	5.8
Signaling by Interleukins	1.35e-05	12	6.1	Interleukin-2 family signaling	1.63e-05	6	19.0	Downstream signaling events of B Cell Receptor BCR	1.41e-04	7	10.7
Activated TAK1 mediates p38 MAPK activation	1.57e-05	5	28.5	Innate Immune System	2.94e-05	28	2.6	Interleukin-2 family signaling	1.96e-04	5	18.8

Pathways ranked based on their significance (Adj. P-value)
