# Supplementary material for: Dynamic Changes in Host Immune Response During Crimean–Congo Hemorrhagic Fever and Severe Fever with Thrombocytopenia Syndrome in Mice
Source: Viruses. 2026 Apr 28;18(5):504. doi: 10.3390/v18050504 (PMC13211718; doi:10.3390/v18050504)
Supplement: Supplementary file 1 [file viruses-18-00504-s001.zip › Supplementary fig.1.pptx]

## Slide 1
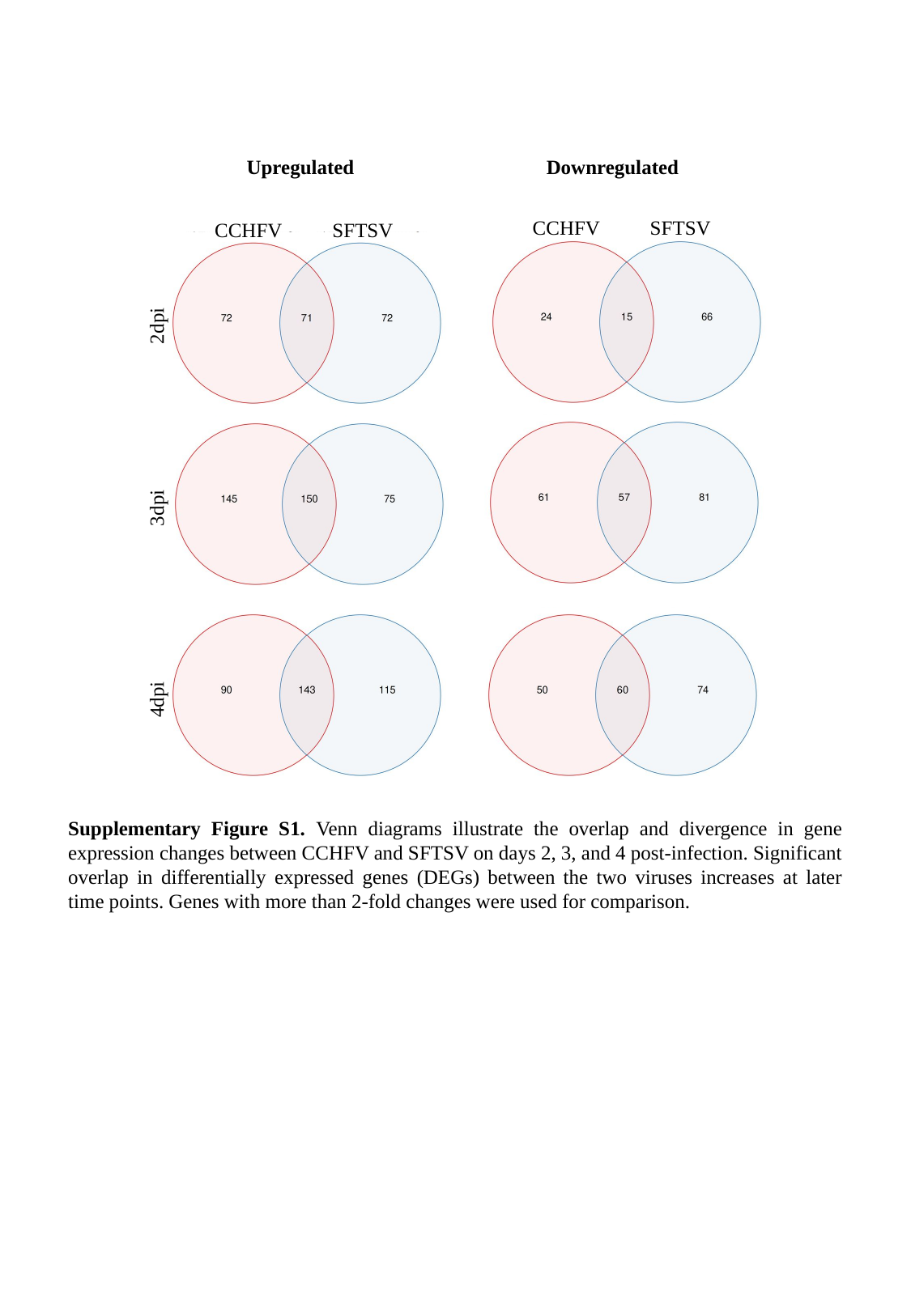

Upregulated
Downregulated
CCHFV
SFTSV
CCHFV
SFTSV
2dpi
3dpi
4dpi
Supplementary Figure S1. Venn diagrams illustrate the overlap and divergence in gene expression changes between CCHFV and SFTSV on days 2, 3, and 4 post-infection. Significant overlap in differentially expressed genes (DEGs) between the two viruses increases at later time points. Genes with more than 2-fold changes were used for comparison.
